# Can Neuromodulation also Enhance Social Inequality? Some Possible Indirect Interventions of the State

**DOI:** 10.3389/fnhum.2017.00113

**Published:** 2017-03-07

**Authors:** Andrea Lavazza

**Affiliations:** Department of Neuroethics, Centro Universitario InternazionaleArezzo, Italy

**Keywords:** NIBS, tDCS, response variability, autonomy, enhancement regulation, neuroethics

## Abstract

There is evidence that noninvasive brain stimulation (NIBS), and especially transcranial direct current stimulation (tDCS), can improve some cognitive functions, at least temporarily. However, as the improvement only applies to some “lucky” people, it may raise ethical, social and legal issues related to fairness in selective contexts (exams, competitions, job interviews). In this regard, an important element tends to be overlooked: the variability in individual response to tDCS in particular. If intensive study or practice and massive doses of chemical enhancers can have slightly different effects over different people, tDCS can sometimes be completely ineffective. The variability in individual response, if tDCS were widely used, could add to the already present natural inequalities between people, or even create new ones, leaving some in a disadvantaged condition. The discussion of the various ethical, social and legal consequences of different individual responses to tDCS might also address a potential indirect intervention by the State. In fact, if NIBS were to be widespread in competitive contexts, those who do not benefit from tDCS would be disadvantaged compared to those able to enhance their skills thanks to neuromodulation technologies. The most disadvantaged people for their lower response to tDCS could then acquire the right to receive and use free and safe cognitive enhancing drugs or other forms of bettering cognitive skills and functions, so as to reduce the gap between them and those who respond well to tDCS, in the light of the principle of equal opportunity.

## Introduction

Noninvasive brain stimulation (NIBS) seems to have recently become the new frontier of enhancement for healthy subjects (Cohen Kadosh, 2014; Dubljević et al., 2014; Bain et al., 2015). Pharmacological interventions aimed at increasing cognitive skills have so far attracted the scholars’ attention (Farah et al., 2004; Greely et al., 2008; Savulescu and Bostrom, 2009; Sahakian et al., 2015), also as regards their ethical, social and legal implications. Indeed, molecules able to influence human executive functions have long been available and arouse questions with respect to their distribution in self-administration without medical supervision. Although there are no reliable data, it is believed that many professionals and college students make use of chemical enhancers (Sahakian and Morein-Zamir, 2007; Vrecko, 2013).

In the last few years, though, there has been growing evidence that NIBS—and especially transcranial direct current stimulation (tDCS), the most used technique—can improve some cognitive functions, at least temporarily, thanks to the effects of neuromodulation, i.e., the facilitation of the excitability and discharge capacity of neurons through weak current (1–2 mA) applied with electrodes placed on the scalp (Chi and Snyder, 2012; Hauser et al., 2013; McKinley et al., 2013; Snowball et al., 2013; Brunoni and Vanderhasselt, 2014; Coffman et al., 2014; Mayseless and Shamay-Tsoory, 2015; Parkin et al., 2015; Podda et al., 2016).

For example, tDCS improved the vigilance and target detection of pilots. Twenty seven Air Force soldiers were stimulated with tDCS (2 mA for 30 min, anode in area F10) and then given the synthetic aperture radar target learning task, consisting of a radar simulator with red or blue circular patterns in which participants had to identify the target stimuli (for example, vehicles or missiles; McKinley et al., 2013). Each target stimulus could appear in different points of space. Each participant received 90 min of preparatory training for the task and a tDCS session. Anodal tDCS produced improvement in the accuracy of the visual search task by about 25% compared to the sham (placebo) group.

This has promptly triggered the reflections of both neuroscientists working with NIBS and ethicists trying to sketch a framework within which to evaluate the enhancement of healthy individuals (Cohen Kadosh et al., 2012; Bikson et al., 2013; Walsh, 2013; Cabrera et al., 2014; Maslen et al., 2014; Davis, 2015; Riggall et al., 2015). However, many rapid developments, also under the pressure of private industries, are changing both the spread of tDCS and, more importantly, its ethical, social and legal implications.

First of all, as regards the spread of tDCS, the so-called do-it-yourself (DIY) approach is contributing to the increase of the individual use of NIBS (Fitz and Reiner, 2013; Jwa, 2015; Maslen et al., 2015; Wexler, 2015). This phenomenon primarily happens through the promotion, on dedicated websites, of the (alleged) potential of tDCS and the sharing of instructions for self-administration according to the desired results. Unlike chemical enhancers, devices for tDCS are free to sell and not subject to medical prescriptions. In addition, their cost is low enough to be accessible to a wide audience. There are also instructions on how to build a device yourself, which is relatively cheap and not particularly difficult for anyone with some degree of technical expertise.

Second, largely due to the experiments carried out by private companies, but also on the basis of scientific experiments published in peer-reviewed journals (Davis, 2013; Sales et al., 2016) the attention has been recently brought to the possibility to enhance athletic performance thanks to tDCS. In particular, it has emerged that some athletes who went to the 2016 Olympics in Rio de Janeiro have been trained also using tDCS to stimulate the motor cortex (Strickland, 2016). According to data published by Halo Neuroscience and reported also by *Nature* (Reardon, 2016), stimulation on some US ski jumpers has improved the athletes’ jumping force by 70% and their coordination by 80%, compared to the sham group. Another research soon to be published in a peer-reviewed journal, recently presented by Lex Mauger of the University of Kent (March 2016), shows that direct current stimulation can reduce the athletes’ subjective fatigue, which affects their performance. Among the published studies, one has achieved a reduction in perceived fatigue and a consequent increase in performance by stimulating the temporal cortex (which has an important role in body awareness and automatic functions) of cyclists (Cogiamanian et al., 2007).

Another study has found that healthy subjects with no previous experience with golf, when stimulated with tDCS, improved implicit motor learning (Zhu et al., 2015). In this study, 25 healthy individuals practiced a golf putting task during a training phase while receiving either real cathodal tDCS stimulation over the left DLPFC area or sham stimulation. The stimulation enhanced golf putting performance. In the authors’ opinion, cathodal tDCS “suppresses verbal working memory activity, which would reduce explicit verbal-analytical engagement in movement control, thereby promoting implicit motor learning” (Zhu et al., 2015).

## Specific Features of DIBS

Despite the controversial data (Brunoni and Vanderhasselt, 2014; Coffman et al., 2014), it therefore seems that different applications of tDCS may have enhancement effects, both on the cognitive side and on the athletic side. This means that NIBS on healthy subjects should be considered a special case compared to other forms of enhancement, especially as regards its social and ethical implications. In fact, with chemical enhancers there is a sharp distinction between doping and cognitive enhancement. Doping in sport is certainly very effective, often involves health risks and almost always has clearly identifiable side effects; but it is a socially stigmatized practice, forbidden by the regulations and severely punished (although some ethicists have proposed arguments to accept it; see Savulescu et al., 2004; Cashmore, 2010). Chemical cognitive enhancement, instead, is certainly less effective (Repantis et al., 2010) and its spread is still almost entirely unregulated. Debate on ethical, social and legal implications of human enhancement is very open, with the two main fronts of bio-libertarians and bio-conservatives (Racine, 2010). The former highlight the helpfulness of enhancers, capable to solve many problems, and are in favor of the unrestricted use of every kind of enhancing means (Caplan, 2004), the latter stress the negative impact on personal achievement and wish for their strict regulation and, sometimes, even their prohibition (Sandel, 2009).

NIBS seems to encompass the possibility of enhancing a subject both cognitively and athletically, even though significant effects are yet to be confirmed through further studies. Although it hasn’t been yet approved as a therapeutic tool by the FDA or other entities entrusted with health authorizations, tDCS is traded freely and no sporting regulations prohibit or limit its use. Only after the news that Olympic athletes have used NIBS did the World Anti-Doping Agency (WADA) decide to monitor the use of tDCS, in order to evaluate whether to include it in the list of treatments forbidden by the International Olympic Committee (IOC; Strickland, 2016). The main general criteria established by IOC to be included in the list are risks for the athlete’s health and potential violation of sportsmanship. tDCS is however considered substantially safe (Bikson et al., 2016).

Furthermore, NIBS has specific features that seem to imply serious ethical consequences:

1. The abovementioned ease of unlimited self-administration: once you have a tDCS device, you can use it as long as you wish. On the contrary, with a few exceptions, one always depends on others for the availability of chemical enhancers.
2. The variability of the effects of the stimulation both in the same individual at different times and, above all, between individuals (to the current state of knowledge). The percentage of non-responders varies according to the protocols in which the stimulation is used, but there is evidence that this is a widespread phenomenon (Datta et al., 2012; Meiron and Lavidor, 2013; Krause and Cohen Kadosh, 2014; López-Alonso et al., 2014; Wiethoff et al., 2014; Chew et al., 2015). Inter-individual variability can lead to problems of equality of opportunity, since the different response to NIBS is linked to features the person cannot intervene on, for example skull thickness, subcutaneous fat levels, cerebrospinal fluid density and cortical surface topography (Horvath et al., 2014).
3. The non-detectability of the use of NIBS on healthy individuals with purposes of cognitive or athletic enhancement (to the current state of knowledge). This does not allow one to implement control measures or bans of the use of NIBS, except in very invasive manners and in particular circumstances (for example, by monitoring athletes long before the race).

Taken together, these three elements contribute to the complexity of the ethical, social and legal implications of NIBS, which therefore requires a wider and better outlined framework. In general, the performance enhancement that can be currently achieved through NIBS seems not to be yet such as to create, under equal initial conditions, very strong differences between an individual “enhanced” thanks to tDCS and a “non-enhanced” one. However, neuromodulation technologies are still young and promise to grow rapidly in both precision and reach. Therefore it is justified to worry about the consequences of the increasing popularization of devices for tDCS for the purpose of enhancing healthy individuals. In fact, this may raise relevant issues related to fairness in competitive (races and athletic competitions) and selective contexts (exams, public competitions, job interviews).

Another theme worthy of consideration is that of safety. It is true that literature has not highlighted any damages or side effects of transcranial current direct stimulation, if not fleeting headaches and rare skin lesions (Frank et al., 2010). Recently, though, a group of authoritative scholars has written an Open Letter regarding DIY users of tDCS (Wurzman et al., 2016). They warn their readers that “stimulation affects more of the brain than a user may think”, since “stimulation extends well beyond the regions beneath the electrodes”. The key points raised by Wurzman et al. (2016) are the following: (1) “Stimulation interacts with ongoing brain activity, so what a user does during tDCS changes tDCS effects”; (2) “Enhancement of some cognitive abilities may come at the cost of others. Stimulating one brain area may improve the ability to perform one task but hurt the ability to perform another”; (3) “Changes in brain activity (intended or not) may last longer than a user may think”; and (4) “Small differences in tDCS parameters can have a big effect. Mild changes in tDcs settings including current amplitude, stimulation duration, and electrode placement can have big and unexpected effects” (see also Davis and van Koningsbruggen, 2013).

In the light of these considerations, and given the likely increase in use of tDCS if its use for sports enhancement will prove to be effective, it seems prudent to pay greater attention (even preventively) to NIBS as a means of enhancing healthy individuals. What’s at stake is not only the users’ safety but also the principle of fair competition (encompassing justice and equity): it is auspicable to avoid that someone might unfairly take advantage of the situation to the detriment of others without good reasons and that someone has a undue burden on her fundamental rights (Lavazza and Garasic, 2017). In this scenario, it is important to analyze the role that can be played by the state, both as a regulator and as a “balancer” (provided that NIBS isn’t prohibited altogether).

## Ethical Implications

The debate on enhancers is very heated and cannot be dealt with extensively here. The positions range from the complete rejection of cognitive enhancers and the proposal to ban them up to their full acceptance and active promotion (Garasic and Lavazza, 2016). Each position, consistent with its own assumptions, gives a specific role to the State as an entity capable of imposing universally applicable rules. Simplifying, bio-conservative positions tend to promote individual effort and the acceptance of one’s natural endowments, entailing the restriction and/or prohibition of enhancers (President’s Council on Bioethics, 2003). Bio-liberal positions, instead, privilege individual freedom and autonomous choice, rejecting the implementation of any general rules (Juth, 2011). These two families of positions have different views of individual flourishing and the goals that a just society should pursue, but both give value to equality of opportunity, fairness and justice.

As for the possible indirect interventions of the State, the first theme to address is that of safety. Faced with the spread of tDCS and the lack of univocal scientific data about the (actual or potential) risks and side effects of an intensive and/or long-term use of tDCS, it is necessary to carry out targeted and thorough studies, both on animal models and on human beings. These studies have little chance of being funded and carried out by university researchers because they are expensive and not very “profitable” in terms of scientific publications. It is even less likely that they should be systematically carried out by private companies involved in the production and trade of devices for tDCS. The State could then promote such studies for public health, either through the structures in charge of ensuring the safety of products for sale or by financing scholars working on tDCS.

Another element to consider, which is what interests us here, is that the use of NIBS for the cognitive and athletic enhancement of healthy subjects could increase the already present natural inequalities between people, or even create new ones, leaving some individuals in a disadvantaged condition. In fact, if NIBS were to be widespread in competitive contexts, those who do not benefit from tDCS would be more disadvantaged compared to those able to enhance their skills thanks to neuromodulation technologies. In this sense, the strong inter-individual variability that seems to characterize NIBS is an additional and important feature due to which the means to counter inequalities are different compared to chemical enhancers. Thus, the use of NIBS seems to require different policies in order to restore the equality of opportunities.

## The Role of the State

How should the State intervene in this case? The answer is not as univocal as in the case of safety. Indeed, for some professionals, cognitive enhancement (and perhaps even athletic enhancement: think of firefighters, but also the police, although with some caution) may be of benefit to the whole society and, given the absence of risk and considering the limited number of people involved, this might even translate in a *duty* of enhancement, as some have argued (Santoni de Sio et al., 2014). Indeed, it has been claimed that people like surgeons, some shift workers, and air traffic controllers could have a moral duty to improve their performance with any tools that become progressively available. And this duty could be enforced by the State itself. A plausible objection to this argument is the rejection of coercion to medical treatment and the general principle that people shouldn’t be used as means, whatever their profession.

However, if this argument may hold for chemical enhancers (where one should still assume a degree of inter-individual variability of effectiveness, as happens for any drug), it is certainly much less convincing for NIBS, which seems to produce changing effects over time both on the same individual and between different individuals. This makes it so that one shouldn’t rely more on, say, enhanced surgeons, because tDCS doesn’t always have the same effects in terms of improved attention and coordination (Garasic and Lavazza, 2015). This would produce an unacceptable discrimination if it were decided to only offer such jobs to those who respond well to NIBS; people unresponsive to NIBS don’t seem comparable to those who, due to serious physical or psychological handicaps, are not allowed to perform surgery, as in the latter case there are objective difficulties that prevent them from practicing their profession.

The problem of potentially increasing inequalities thus remains, both due to the different ability to access to NIBS (despite its easy use and low cost) and, mostly, due to the intra- and inter-individual variability of its effects. In this case, if the state is willing to intervene, it seems to have two options: either to ban NIBS or to implement policies of “re-balancing”, as we have suggested earlier. In fact, one could object that the state should do nothing in this field and let things go unaffected. This option is consistent both with the view that tDCS is not and will not be a social and legal problem at all, and with the view that the state should not regulate people’s personal choice to enhance themselves, once it is proven that safety is guaranteed. Nevertheless, if one recognizes that the widespread use of tDCS can give rise to a problem of inequality and that the state has a duty to intervene, one has to consider the two abovementioned options.

The ban hypothesis can be rejected due to the importance, in Western societies, of the principle of individual autonomy: the subject must be free to choose (hopefully with full competence and information) their goals and how to reach them, so long as the freedom of others is also respected (Lavazza, forthcoming). In this sense, the assessment of the safety of NIBS, also promoted by the state, would allow the individual to make an informed choice, knowing whether or not they would face any risks. It can also be argued that the diffusion of enhancements (especially cognitive ones) would benefit society as a whole, with consequences, of varying significance, for all. In fact, it is not implausible to think that the growth of cognitive abilities would lead to an increase of the collective expertise available and facilitate the smooth functioning of the institutions and services that are open to all. However, there is still the crucial problem of fairness and equality of opportunity between citizens. As is known, inequalities in income and wealth, social standing and ability to improve one’s condition constitute one of the most corrosive factors of individual welfare and social relations and, ultimately, of society as a whole (Sen, 1992; Deaton, 2013; Atkinson, 2015).

What the state could do then, as for the enhancers considered here, is allow those with low response to tDCS to use other free and safe cognitive enhancing drugs or different forms of cognitive and/or motor augmentation (more effective for that individual) to improve cognitive skills and functions (specific courses, guided exercises), so as to reduce the gap between them and those who respond well to tDCS, in the light of the principle of equal opportunity. Another form of public intervention could be to subsidize research in neuromodulation technologies and other forms of artificial enhancement, so that fewer people would be excluded from cognitive enhancement effects because of their particular physical constitution.

In this way the State would not play the role of a direct regulator, intervening through specific regulations or the prohibition of NIBS (unless health risks were to emerge from further studies), but simply that of a “balancing” factor. It should also be underlined that prohibiting NIBS would be at least partially ineffective, since it’s currently impossible to determine a subject’s use of that specific enhancement. As a result, the only way to effectively enforce the ban on the use of brain enhancement through stimulation devices, even in specific areas such as high-level selective contexts (like competitions for management positions), would be to isolate the candidates for a long time in controlled environments.

Rather than limiting the use of NIBS, the State could therefore implement an indirect intervention aimed at correcting potential imbalances due to the spread of cerebral enhancers whose selective effects may end up increasing inequality between citizens in terms of cognitive and athletic skills. In particular, supporting the research on efficient and egalitarian enhancers would be an important contribution to the welfare of society.

Obviously, it should be specified that, at least for some time, the only fields affected would likely be very competitive specific areas of society involving a limited number of people. However, despite their relative specificities, these spheres are very delicate because a growing number of people are using enhancement of various kinds, often at the price of significant side effects. In this case, the balancing role of the State, in some situations, would be complicated by the fact that (say) the candidate for a certain position or a top athlete may have already made use of all available enhancers: in this case, there would be no way for the State to compensate for the different starting positions other than to attribute a sort of handicap to those who have a greater benefit from enhancers thanks to their physical constitution. In other words, in selective-competitive contexts, the candidates’ performance rankings should be corrected *ex post*, at least in part, so as to protect an actual equality of opportunity. In this way, implicitly, one would sacrifice efficiency for justice and fairness.

Of course, my aim has been to outline the problem and propose a general solution rather than exposing in detail a specific intervention. The latter task indeed would require much more space and research. Nevertheless, it is easily conceivable that the use of enhancers will spread in society beyond those specific areas, to the point of having to implement the compensatory and rebalancing measures proposed here.

## Author Contributions

The author confirms being the sole contributor of this work and approved it for publication.

## Conflict of Interest Statement

The author declares that the research was conducted in the absence of any commercial or financial relationships that could be construed as a potential conflict of interest.
